# Prevalence of *SCN1A*-Related Dravet Syndrome among Children Reported with Seizures following Vaccination: A Population-Based Ten-Year Cohort Study

**DOI:** 10.1371/journal.pone.0065758

**Published:** 2013-06-06

**Authors:** Nienke E. Verbeek, Nicoline A. T. van der Maas, Floor E. Jansen, Marjan J. A. van Kempen, Dick Lindhout, Eva H. Brilstra

**Affiliations:** 1 Department of Medical Genetics, University Medical Center Utrecht, Utrecht, The Netherlands; 2 Center for Infectious Disease Control, National Institute for Public Health and the Environment, Bilthoven, The Netherlands; 3 Department of Child Neurology, Rudolf Magnus Institute of Neuroscience, University Medical Center Utrecht, Utrecht, The Netherlands; Pasteur Institute of Lille, France

## Abstract

**Objectives:**

To determine the prevalence of Dravet syndrome, an epileptic encephalopathy caused by *SCN1A-*mutations, often with seizure onset after vaccination, among infants reported with seizures following vaccination. To determine differences in characteristics of reported seizures after vaccination in children with and without *SCN1A*-related Dravet syndrome.

**Methods:**

Data were reviewed of 1,269 children with seizures following immunization in the first two years of life, reported to the safety surveillance system of the Dutch national immunization program between 1 January 1997 and 31 December 2006. Selective, prospective follow-up was performed of children with clinical characteristics compatible with a diagnosis of Dravet syndrome.

**Results:**

In 21.9% (n = 279) of children, a diagnosis of Dravet syndrome could not be excluded based on available clinical data (median age at follow-up 16 months). Additional follow-up data were obtained in 83.9% (n = 234) of these children (median age 8.5 years).

15 (1.2% of 1,269; 95%CI:0.6 to 1.8%) children were diagnosed with *SCN1A*-related Dravet syndrome. Of all reported seizures following vaccinations in the first year of life, 2.5% (95%CI:1.3 to 3.6%) were due to *SCN1A*-related Dravet syndrome, as were 5.9% of reported seizures (95%CI:3.1 to 8.7%) after 2^nd^ or 3^rd^ DTP-IPV-Hib vaccination.

Seizures in children with *SCN1A*-related Dravet syndrome occurred more often with a body temperature below 38.5°C (57.9% vs. 32.6%, p = 0.020) and reoccurred more often after following vaccinations (26.7% vs. 4.0%, p = 0.003), than in children without a diagnosis of *SCN1A*-related Dravet Syndrome.

**Conclusions:**

Although Dravet syndrome is a rare genetic epilepsy syndrome, 2.5% of reported seizures following vaccinations in the first year of life in our cohort occurred in children with this disorder. Knowledge on the specific characteristics of vaccination-related seizures in this syndrome might promote early diagnosis and indirectly, public faith in vaccination safety.

## Introduction

Due to the success of national immunization programs, life-threatening and disabling complications of many infectious diseases have been forgotten. Fear of alleged side-effects of vaccinations can lead to a decrease in vaccination coverage, inadequate herd immunity and subsequent epidemics of infectious diseases. Open and transparent information on side-effects of vaccinations is needed to consolidate adequate vaccination coverage.[1], [2] One of the most frightening side-effects of vaccinations might be febrile seizures. Epidemiological studies show an increased risk of febrile seizures of 25 to 34 per 100,000 Measles Mumps Rubella (MMR) vaccinations,[3] six to nine additional cases per 100,000 Diphtheria Tetanus whole cell Pertussis (DTwP) vaccine doses,[3], [4] and no or only mild increased risk after Diphtheria Tetanus acellular Pertussis (DTaP) vaccine.[5] Vaccination is not associated with an increased risk of afebrile seizures, epilepsy or irreversible neurological damage.[5], [6] However, it cannot be excluded that vaccinations might trigger seizures in certain subgroups of children with a high seizure susceptibility. In 2006, Berkovic et al, showed that among a case series of children in whom vaccination had been implicated as a direct cause of the subsequent epileptic encephalopathy, 11 out of 14 children had Dravet syndrome due to *SCN1A-*mutations.[7] This showed that the epilepsy and retardation in these children was primarily due to a genetic defect and not to the vaccination.

Dravet syndrome, also known as Severe Myoclonic Epilepsy of Infancy (SMEI), is a rare epilepsy syndrome (estimated prevalence 1∶20,000–40,000) [8]–[10] with onset in the first year of life in previously healthy children. After, often prolonged, febrile seizures or hemiconvulsions, therapy resistant afebrile seizures and neurological deterioration occur.[11] In at least 70% of children, Dravet syndrome is caused by a heterozygous mutation in the *SCN1A-*gene, [10], [12] encoding the alpha-subunit of the neuronal voltage gated sodium channel. Of these mutations, 95% are spontaneous (‘de novo’) mutations.[13], [14] In a small proportion of children with Dravet syndrome mutations in other genes have been identified as the cause of the epilepsy.[15] Seizures in children with Dravet syndrome are often triggered by fever or infectious diseases. Vaccinations precede first seizures in 7–57% of children with Dravet syndrome.[10], [16]–[19] Although Dravet syndrome is a rare disorder, among children with seizures following vaccinations the prevalence might be significant. Information on the prevalence and characteristics of early, vaccination-related seizures in Dravet syndrome, is of major importance for recognition of Dravet syndrome among children presenting with seizures following vaccinations. An early diagnosis of Dravet syndrome is important for treatment (avoidance of sodium channel blockers) [20] and genetic counseling. The knowledge that vaccination, although possibly the trigger for the first seizure, is not the cause of their child's genetic epilepsy syndrome could support immunization of other children in the family.

Our study comprises a 10-year cohort of children with seizures following vaccinations in the first two years of life, reported to the enhanced passive safety surveillance system for the Dutch national immunization program. Within this cohort, children with possible Dravet syndrome were followed up, to estimate the prevalence of *SCN1A*-related Dravet syndrome and describe the clinical characteristics of seizures following vaccinations.

## Methods

### Ethics Statement

This study was conducted in accordance with the Declaration of Helsinki and approved by the Medical Ethics Committee of the University Medical Centre Utrecht, Utrecht, the Netherlands (No 07/295).

### Setting: safety surveillance of immunizations

The Dutch national immunization program is a voluntary program with stable and high vaccination coverage (∼95%).[21] The vaccination registry is linked to the population register and records all vaccinations at an individual level. Safety surveillance is performed by an enhanced passive reporting system, that was carried out by the National Institute for Public Health and Environment (RIVM) until 2011.

Adverse events following immunization (AEFI) were reported and registered in a database after extensive supplementation and verification. In case of serious or uncertain diagnoses and unresolved events, follow-up was done. Reporting rate was stable and high.[22] Causal relation of reported seizures with the vaccination was assessed routinely. Seizures occurring within 24 hours following administration of an inactivated vaccine (DTP-IPV, Hib or MenC),[3] or 5 to 12 days after MMR vaccination,[23] were considered to be causally related to vaccination. Temperatures were categorized as <37.5, 37.5–38.4 or ≥38.5°C.

### Study population and vaccination schedules

The total study population comprised all children with adverse events after a vaccination in the first two years of life, reported to RIVM between 1 January 1997 and 31 December 2006.

During this period the schedule included four doses of Diphtheria, Tetanus, Pertussis, inactivated Polio vaccine and Haemophilus influenzae type b (DTP-IPV(-)Hib) in the first year of life and one dose of MMR vaccine at age 14 months. Hepatitis B (HepB), Pneumococcal conjugate vaccine (PCV), Meningococcal serogroup C (MenC) and acellular Pertussis (aP) were introduced between 2001 and 2006 (see Table S1).[22], [24], [25]

### Design of study

**Figure 1:**
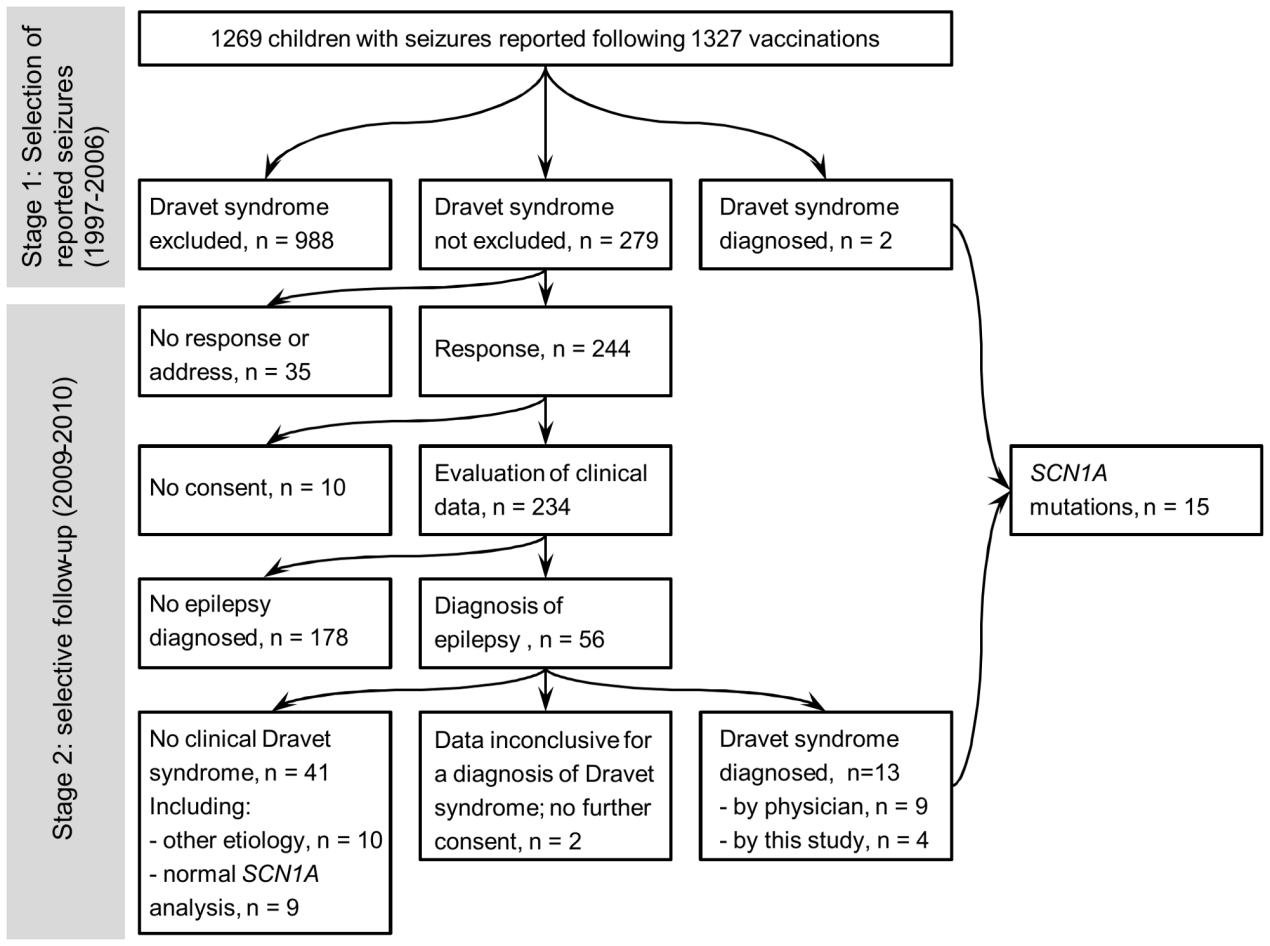
Design of study.

Figure 1, schematically represents the design of the study. In stage 1 we prospectively selected all children with reported ‘fits’ (convulsions, atypical seizures, epilepsy), ‘myoclonus’, ‘retardation’, ‘encephalopathy/encephalitis’ or ‘death’ reported during the studied period.[22] All events, regardless of their relation to vaccination, were included if they could be classified as a febrile seizure, an afebrile seizure or an atypical seizure (see ‘classification of seizures’). Reports of collapse, defined by sudden onset of pallor, limpness and hyporesponsiveness, were excluded.[26] From the RIVM database and from hard copy files, patient characteristics were collected including the ages at first seizure, last seizure and last follow-up, types of seizures, psychomotor development, comorbidity and etiology. In children diagnosed with epilepsy, data on response to treatment and results of electroencephalography (EEG) and brain scans were collected as well.

At stage 2, we contacted parents of children who's medical history met the criteria for a clinical diagnosis of Dravet syndrome (see ‘case definitions’), or in whom Dravet syndrome could not be excluded on the basis of the data available at stage 1. Parents were asked to give written consent to obtain all relevant data from medical records. They could also provide follow-up data themselves.

The medical records were reviewed to classify the data as possibly compatible with a diagnosis of Dravet syndrome by an experienced pediatric neurologist (FJ) and clinical geneticist (NV), independently. In case of disagreement, a second clinical geneticist (EB) also reviewed the data and consensus was reached between the three.

In children eventually diagnosed with *SCN1A*-related Dravet syndrome, the complete vaccination report was obtained from the vaccination registries.

### DNA analysis of the SCN1A-gene

DNA-analysis of the *SCN1A*-gene was performed if clinical criteria for Dravet syndrome were met according to the treating physician or the study group. Parents gave informed consent for the DNA analysis. DNA-analysis of the *SCN1A*-gene included sequence and MLPA analysis, as previously described.[27] All cases were analyzed by the laboratory for DNA diagnostics at the UMC Utrecht, except for one case who had been analyzed by the DNA Diagnostic Unit of the Neurogenetics Group, VIB Department of Molecular Genetics, University of Antwerp, Belgium.

A detected mutation was considered to be pathogenic when it had been described earlier in Dravet syndrome patients, when it was predicted to lead to a truncated protein or haploinsufficiency, or when the mutation was not detected in the parents and therefore assigned as ‘de novo’ in the patient.

### Statistical analysis

The outcome of interest was the proportion of seizures attributed to *SCN1A*-related Dravet syndrome among all reported seizures after vaccination in the first two years of life, and later categorized per year and per vaccination type. These proportions, were also calculated for the subgroups of ‘vaccination-related’ seizures and seizures reported within two months following vaccination, in order to assess the influence of delayed notifications and of seizures unrelated to vaccinations as confounders. 95% confidence intervals (95%CI) were calculated.

Differences in characteristics at stage 1, between children diagnosed with *SCN1A*-related Dravet syndrome and all other children reported with seizures, were analyzed. We used the Mann-Whitney U test to compare the two groups with respect to ages at vaccination, first seizure, notification of event, and follow-up. Differences in proportions of vaccination type (DTP-IPV or MMR), and of causality of vaccination to seizure, type of seizure (atypical or not) and temperature (< or ≥38.5°C), categorized per vaccination type, were calculated with either Chi-Square analysis, or Fisher's Exact Test.

All statistical analyses were performed using SPSS Statistics, release 17.0 or 20, except for calculation of 95%CI (Excel).

### Case definitions

#### 1. Classification of seizures (stage 1 of study)

All events with tonic and/or clonic muscle spasms and loss of consciousness had been classified as seizures. They were further classified as afebrile, febrile (simple and complex), or atypical. Seizures occurring with a temperature of 38.5°C or above, and those with substantial rise of body temperature but without temperature recorded, were classified as febrile seizures. Febrile seizures that were prolonged (>15 minutes), asymmetrical or recurred within 24 hours, were classified as complex febrile seizures.

Seizures were classified as atypical when the description of the seizure characteristics was insufficient for a definite classification as an epileptic seizure, febrile or afebrile. Atypical seizures occurring multiple times within 24 hours were classified as one episode of atypical seizures.

#### 2. Selection of children with possible Dravet syndrome (stage 1 of study)

Dravet syndrome was considered in all children diagnosed with epilepsy younger than 24 months and less than eight months seizure-free at follow-up in stage 1, and a course suggestive of Dravet syndrome (see ‘clinical criteria for Dravet syndrome’).

In the other children a diagnosis of Dravet syndrome was considered, if the report consisted of:

- a single seizure before twelve months of age, and a follow-up of less than six months after the seizure;
- an afebrile or complex febrile seizure before age 18 months and a follow-up of less than three months after the seizure;
- multiple simple febrile seizures before age 12 months or multiple other seizures before age 18 months, and less than six months of follow-up after the last seizure;
- one or more atypical seizures, fulfilling one of the former three criteria with respect to age at time of seizures and follow-up.

#### 3. Exclusion of Dravet syndrome in children (stage 1 and 2 of study)

Dravet syndrome was excluded in children:

- with epilepsy of other etiology, or with electroclinical syndromes other than Dravet syndrome (i.e. West syndrome);
- with a severe developmental delay before epilepsy onset;
- not diagnosed with epilepsy by the age of three years or older.

#### 4. Clinical criteria for Dravet syndrome

Dravet syndrome was diagnosed in children with [11]:

- seizure onset within the first 12 months of life;
- normal development before onset of seizures;
- generalized tonic-clonic seizures or unilateral seizures as initial seizure type;
- normal EEG in first year of life;
- development of epilepsy with multiple seizure types, with or without myoclonus, after the first year of life;
- a (temporarily) drug-resistant epilepsy;
- slowing of development after first year of life;
- exacerbation or provocation of seizures with hyperthermia; *and*
- normal brain imaging or aspecific abnormalities only.

When only one criterion was not fulfilled, Dravet syndrome was still diagnosed.[28]

## Results

### Study population

Within the 10-year cohort, 1,269 children (681 males; 53.7%) had been reported with seizures following 1,327 vaccinations in the first two years of life. 4.2% (n = 54) of children had reported seizures after two (n = 50) or three (n = 4) different vaccination moments (Table 1). Of all reported AEFI in this 10-year cohort, 10.5% were classified as seizures (1,327 out of 12,634), i.e. a reporting rate of seven per 10,000 vaccinated children.

**Table 1:** Baseline characteristics of children with seizures following vaccinations, and with or without *SCN1A*-related Dravet syndrome.

		All children reported (n = 1269)		P-value	Total number
Characteristics of reports		*SCN1A*-related Dravet syndrome (n = 15)	No Dravet syndrome diagnosed (n = 1254)		
Number of males		10 (66.7%)	671 (53.5%)	0.310	1269
Median age in months (range), at	first seizure	4 (3–6)	11 (0–46)*	0.001	1259
	administration of first reported vaccine	4.4 (2.8–12.2)	11.5 (0.2–23.9)	0.001	1269
	notification of seizure after vaccination to RIVM	5.4 (3.9–165.9)	14.4 (0.3–174.5)	0.070	1269
	end of follow-up (stage 1)	18 (4–166)	16 (0–175)	0.619	1269
Number of children of whom first reported seizure was	vaccination-related	14 (93.3%)	956 (76.2%)	0.216	1269
	first seizure	13 (86.7%)	1157 (92.3%)	0.329	1269
	first seizure ànd vaccination-related	12 (80.0%)	862 (68.7%)	0.416	1269
Number of children with	multiple notifications of seizures after vaccination	4 (26.7%)	50 (4.0%)	0.003	1269
	multiple seizures, during follow-up (stage 1)	14 (93.3%)	375 (29.9%)	<0.001	1269
	diagnosis of epilepsy (stage 1)	10 (66.7%)	87 (6.9%)	<0.001	1269

Median age at first seizure was missing in 10 children. These were seizures unrelated and prior to, the reported vaccination.

* 4 children with first seizure after age 2.0 years, and at least 0.5 years after last vaccination.

**Figure 2:**
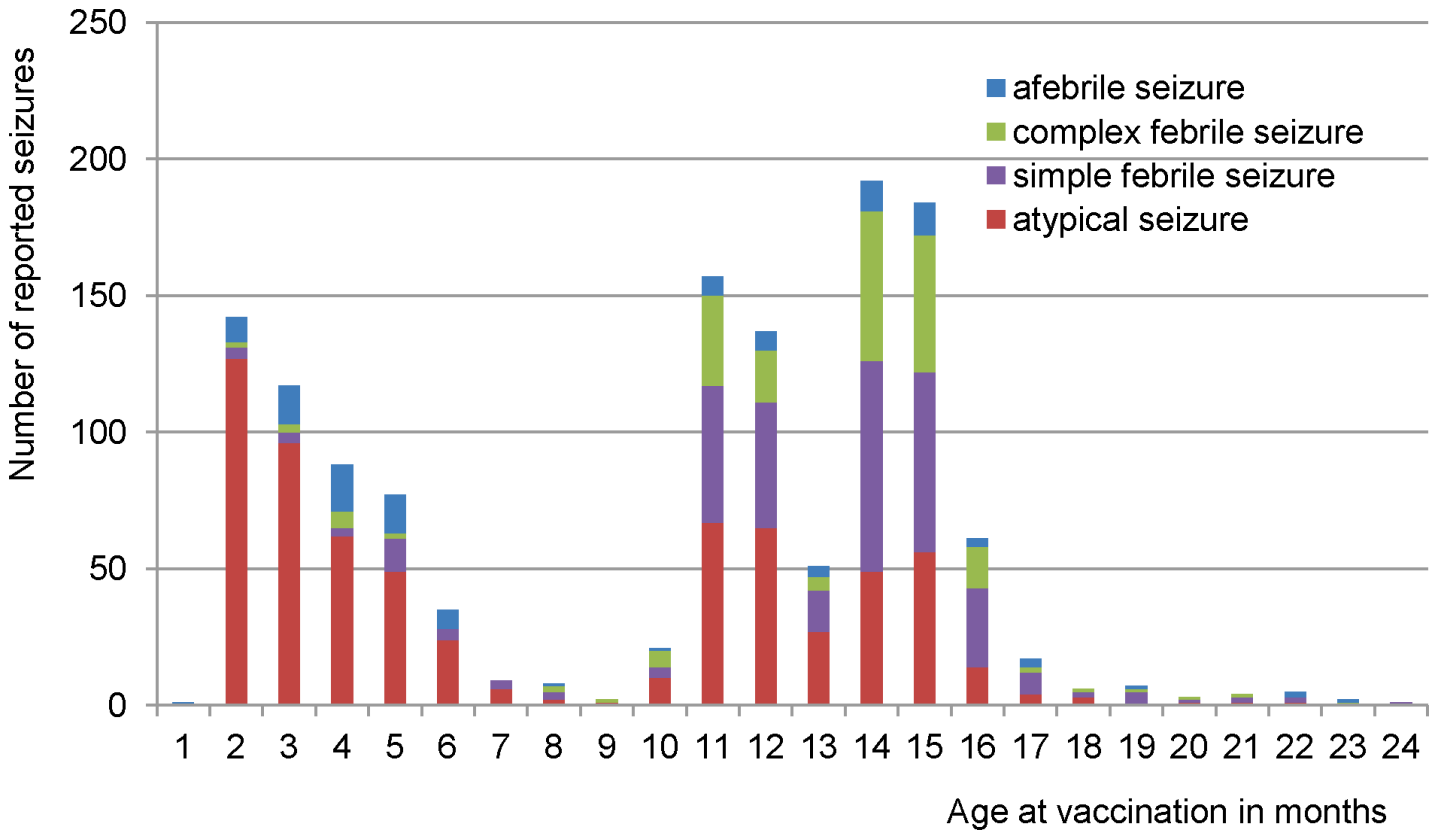
Classification and distribution of reported seizures following vaccinations, according to age at vaccination.

The reported vaccinations (n = 1,327) included 64.5% (n = 856) DTP-IPV(-)Hib vaccinations, 33.2% (n = 440) MMR (+MenC) vaccinations, 1.2% (n = 16) combined DTP-IPV(-)Hib/MMR vaccinations and 1.1% (n = 15) other vaccinations (mainly single MenC vaccinations). 55.2% (n = 732) of reported seizures occurred after a vaccination in the first year of life. Of the reported seizures 50.1% (n = 665) were atypical seizures, 25.7% (n = 341) simple febrile seizures, 15.5% (n = 206) complex febrile seizures and 8.7% (n = 115) afebrile seizures (Figure 2).

In 76.3% (n = 1,013) of the reported seizures a possible causal relation with the vaccination was assessed.

The median age at vaccination, was 11.5 months (m) (range 0.2 m–23.9 m). The median time between vaccination and report of AEFI was 1.8 m (range 0 days–14.1 years (y)). The median age at end of follow-up of stage 1 was 16 m (range 0 m–14.6 y). At that moment, 30.7% (n = 389) of children had had more than one seizure, including 7.8% (n = 99) with seizures preceding the reported AEFI, and 7.6% (n = 97) of children had been diagnosed with epilepsy (Table 1).

### Case ascertainment in total cohort (stage 1; n = 1,269 children)

In the total cohort, two children already had been diagnosed with Dravet syndrome by the treating physician, before the end of follow-up at stage 1.

In 21.9% (n = 279) of children, a clinical diagnosis of Dravet syndrome could not be excluded, of whom 43 children diagnosed with epilepsy.

In 2.0% of children (n = 25) a different etiological diagnosis was made. In the other 75.9% (n = 963) of children, a clinical diagnosis of Dravet syndrome was excluded (Figure 1, Table S2).

### Identification of children with Dravet syndrome within the selected subcohort (stage 2; n = 279)

Follow-up information was obtained for 83.9% (n = 234) of children in whom Dravet syndrome was still considered possible (Figure 1, Table S3). The median age at follow-up at stage 2 was 8.5 y (range 3.1–23.6 y).

Sixteen children had been diagnosed with epilepsy after stage 1, bringing the total number of children diagnosed with epilepsy to 56 (23.9% of 234). Of them, nine had already been diagnosed with clinical Dravet syndrome by their treating physician and pathogenic *SCN1A*-mutations had been detected in eight of them (Figure 1, Table 3, Table S3). By means of this study, a *SCN1A*-mutation has been detected also in the ninth child. Based on available clinical data, we additionally diagnosed Dravet syndrome in four children, and detected pathogenic *SCN1A*-mutations in all four. Of these 13 children with *SCN1A*-related Dravet syndrome, eight had already been diagnosed with epilepsy at stage 1 of the study. Of the other five, four had had multiple seizures and one was reported with a single seizure to the RIVM (Table S2).

**Table 3:** Description of seizures following vaccinations in children with *SCN1A*-related Dravet syndrome.

Number	M/F	vaccination	Age at vaccination, in months	First seizure?	Seizure related to vaccination?	Time between vaccination and seizure	Delay in notification to RIVM	Classification of seizure, at time of notification	Description of seizure	Duration of seizure , in minutes	Temperature (°C)	*SCN1A* -mutation	Inheritance
1	M	DTwP-IPV2 Hib2	4.9	Yes	Yes	<24 h	6.5 years	Complex FS	GTCS	15*	38.6	p.Asp1239Tyr	De novo
2	M	DTwP-IPV-Hib2	3.7	Yes	No	17 days	15 months	Complex FS	GTCS	>30	38.5–39	p.Leu1717Pro	De novo
3	F	DTwP-IPV2 Hib2	3.9	Yes	Yes	8 h	7 weeks	Afebrile	GTCS	30*	‘no’	p. Phe1535fs	ND
		DT-IPV4 Hib4	12.4	-	Yes	8.5 h	1 week	Afebrile	‘Seizure’	?	37.8		
4	F	DTwP-IPV1 Hib1	3.4	Yes	Yes	5 h	4 weeks	Complex FS	GTCS	30*	39	p.Gly1433Glu	De novo
		DTwP-IPV2 Hib2	5.5	-	Yes	7 h	2 days	Afebrile	Focal	6*	38.1		
						18 h			Focal	*	37.5		
		DTwP-IPV3 Hib3	6.9	-	Yes	5 h	4 weeks	Simple FS	GTCS	15*	39		
5	M	DTwP-IPV2 Hib2	4.2	Yes	Yes	7 h	2 weeks	Atypical	GTCS?	15	38.3	p.Arg946His	ND
						8 h			Focal	<2			
6	M	DTwP-IPV Hib2	3.1	Yes	Yes	4 h	4 weeks	Atypical	GTCS?	15	38.5	p. Arg101Gln	De novo
7	M	DTwP-IPV3 Hib3	4.4	Yes	Yes	4.5 h	4 days	Afebrile	GTCS	15	‘no’	duplication exon 17–20	De novo
		DTwP-IPV-Hib4	11.7	-	Yes	6 h	1 week	Afebrile	GTCS	5–10	‘no’		
8	M	DTwP-IPV3 Hib3	4.8	Yes	Yes	8 h	3 days	Atypical	Hemi	5	‘warm’	c.4278_4282delCAAGT	ND
						13 h			GTCS	10	>38		
						14 h			‘Seizure’	?			
9	F	DTwP-IPV3 Hib3	4.6	Yes	Yes	11.5 h	4 months	Simple FS	GTCS	10–15	38.9	p.Val1390Met	De novo
10	F	DTwP-IPV-Hib2	3.5	Yes	Yes	10 h	2 weeks	Atypical	Focal	<10	‘no’	c.3880-1G>A**	P Mosaic**
		(DTwP-IPV-Hib3***	4.5	-	Yes	<24 h	1 day	*Not recognized*	GTCS	<15	‘no’)		
11	M	DTwP-IPV-Hib2	2.8	Yes	Yes	6 h	1 month	Afebrile	GTCS	30	38.3	p.Trp384Arg	De novo
		DTwP-IPV-Hib3	4.0	-	Yes	10.5 h	1 day	Afebrile	GTCS	60	38.4		
12	M	DTwP-IPV2	4.4	Yes	Yes	8.5 h	13 years	Simple FS	GTCS	6–7	38.7	p.Ser1879fs	De novo
13	M	DT-IPV4 Hib4	12.2	No	Yes	21 h	3 months	Simple FS	GTCS	2–3	NA	p.Gly177Glu	ND
14	F	DTwP-IPV3 Hib3	6.9	No	Yes	12 h	1 day	Afebrile	GTCS	5–7*	∼38	p.Leu331fs	ND
15	M	DTwP-IPV3 Hib3	5.1	Yes	Yes	5 h	1 week	Afebrile	GTCS	30	37	c.2176+2T>A	De novo

Children 1–2 had been diagnosed at stage 1 and children 3–11 at stage 2 of the study by their treating physician. Children 12–15 were diagnosed at stage 2, by means of this study.

DTwP-IPV-Hib = Diphtheria, Tetanus, whole-cell Pertussis, inactivated Polio vaccine, Haemophilus influenzae type b; FS = febrile seizure; GTCS = Generalized tonic clonic seizure; ND = not determined; h = hours. NA = not available.

* Use of valium rectiole or intravenous;

** DNA diagnostics performed by DNA Diagnostic Unit of the Neurogenetics Group, VIB Department of Molecular Genetics, University of Antwerp; unaffected parent mosaic for mutation.

*** Seizure was reported to the RIVM, but was not recognized as a seizure by either parents or co-workers of the RIVM.

In two children with epilepsy the clinical criteria for Dravet syndrome were partly met, but no further consent for data retrieval or DNA-analysis was obtained.

In the children without a clinical diagnosis of Dravet syndrome (41 with epilepsy, 178 without epilepsy), 15 (10 with epilepsy) had a different etiological diagnosis (including two with a milder form of *SCN1A*-related seizures) and in 12 (9 with epilepsy) *SCN1A*-analysis had been requested by the treating physician and had shown normal results. Dravet syndrome was not further considered in patients without a diagnosis of epilepsy.

### Percentage of children with SCN1A-related Dravet syndrome

In total, 15 children (1.2% of the total cohort (n = 1269), 95%-confidence interval (CI):0.6 to1.8%) were diagnosed with *SCN1A*-related Dravet syndrome (Table 1, Figure 1).

2.5% (n = 18; 95%CI:1.2 to 3.4%) of all reported seizures in the first year of life had occurred in children with *SCN1A*-related Dravet syndrome. In children with Dravet syndrome seizures following vaccination both occurred at a younger age (median age of 4.4 m vs. 11.5 m in all other children, p = 0.001), and were more frequently reported after subsequent vaccinations (26.7% vs. 4.0% in all other children, p = 0.003). After the second and third DTP-IPV(-)Hib vaccination the frequency of seizures due to Dravet syndrome was the highest: 5.9% (n = 16; 95%CI:3.1 to 8.7%).

Only two seizures in children with *SCN1A*-related Dravet syndrome occurred after vaccinations in the second year of life, representing 0.3% (95%CI:0.0 to 0.8%) of reported seizures in the second year of life.

The delay of notification of the seizure to the RIVM was several years in two children with Dravet syndrome (case 1 and 12) (Table 3). In one child with Dravet syndrome there was no relation in time between occurrence of the seizure and administration of the vaccine (case 2). When both reports with a delay of two months or more, as well as seizures unrelated to vaccinations were excluded as potential confounders (n = 553 seizures left), the percentage of seizures attributed to Dravet syndrome was 0.5% (95%CI:0.0 to 1.5%) for the second year, 3.9% (95%CI:1.9 to 5.9%) for the first year and 8.7% (95%CI:4.0 to 13.4%) for the second and third vaccination.

No children with Dravet syndrome were detected in the last two cohort years, in which whole-cell pertussis was replaced by acellular pertussis vaccine. However, there was no statistically significant difference in the proportion of children with Dravet syndrome in the last two years compared with the first eight cohort years (0/59 vs. 20/797 reports on children with Dravet syndrome per reported DTP-IPV(-)Hib vaccination, p = 0.390).

### Vaccination and seizure characteristics in children with SCN1A-related Dravet syndrome

Of the children diagnosed with *SCN1A*-related Dravet syndrome, ten were males (66.7%; p = 0.31). The median age of the first seizure was 4 m (range 3 m–6 m) (vs. 11 m, in all other children, p = 0.001)(Table 1). In 80.0% (n = 12) the first seizure had been vaccination-related (vs. 68.7% of all other children, p = 0.416) and had occurred at a mean age of 4.1 months (Table 3).

All 20 reported seizures occurred 4 to 21 hours (h) (median 7.5 h) after DT(P)-IPV(-)Hib vaccinations, except for one seizure unrelated to vaccination (17 days in case 2) (Table 3). One reported seizure had not been recognized as a seizure by the parents or the co-workers of the safety surveillance system (case 10, Table 3). In two subjects, seizures occurred following MMR vaccinations, but were not reported to the RIVM (data not shown).

Body temperature during seizures was often below 38.5°C (57.9% in children Dravet syndrome vs. 32.6% in all other children, p = 0.020; only DTP-IPV(-)Hib vaccinations analyzed). The reported seizures were generalized tonic-clonic seizures, hemiconvulsions and focal seizures. 15.0% of seizures recurred on the same day. Duration of seizures was two to 60 minutes. In 30.0% they lasted more than 15 minutes. Seizures in children with *SCN1A*-related Dravet syndrome were classified as afebrile (45.0%), simple febrile (20.0%), complex febrile (15.0%) or atypical seizures (20.0%). Atypical seizures were less frequently reported than in other children (20.0% vs. 64.1%; p<0.001; only DTP-IPV(-)Hib vaccinations analyzed) (Table 2 and 3).

**Table 2:** Characteristics of reported seizures following DTP-IPV(-)Hib vaccination in children with and without *SCN1A*-related Dravet syndrome.

	All reported seizures (n = 1327), in children with		P-value	Total number
Number of reported seizures	*SCN1A*-related Dravet syndrome (n = 20)	No Dravet syndrome diagnosed (n = 1307)		
after DTP-IPV(-)Hib vaccination (vs. MMR, combined or other vaccines)	20 (100.0%)	836 (64.0%)	<0.001	1327
2^nd^ or 3^rd^ DTP-IPV(-)Hib vaccination	16 (80.0%)	255 (30.5%)	<0.001	856
vaccination-related	19 (95.0%)	637 (76.2%)	0.059	856
classified as atypical seizure (vs. febrile or afebrile seizure)	4 (20.0%)	536 (64.1%)	<0.001	856
temperature <38.5°C	11 (57.9%)	250 (32.6%)	0.020*	787

* P = 0.046 when the 69 missings were considered as temperature less than 38.5°C.

All children with *SCN1A*-related Dravet syndrome had developed multiple seizure types that were temperature-sensitive. Epilepsy was refractory to treatment in all patients, except in case 9, who was seizure-free with use of anti-epileptic drugs, and in case 13, who only had a few seizures per year. In the first year of life, all children had a normal development. Most children had a moderate to severe developmental delay at stage 2. Case 7 had only mild learning difficulties.

In 86.7% (n = 13) of children, the vaccination schedule was adjusted. Three children received no further vaccinations. In the other children, either one or more subsequent vaccinations did not include the pertussis component (n = 4), some vaccination moments were missed (n = 5) or both (n = 1). (Table S3)

## Discussion

In our nation-wide ten-year cohort study, we identified *SCN1A*-related Dravet syndrome as the underlying cause in 1.2% of children reported with seizures following vaccinations in the first two years of life, including 2.5% of seizures reported after vaccination in the first year of life, and 0.3% in the second year of life. Among seizures reported after the second or third DTP-IPV(-)Hib vaccination, the proportion of *SCN1A*-related Dravet syndrome was the highest.

The seizure had occurred within 24 h (median 7.5 h) after administration of DT(P)-IPV(-)Hib vaccines in the majority of children diagnosed with *SCN1A*-related Dravet syndrome.

Children diagnosed with *SCN1A*-related Dravet syndrome had a younger age at first seizure following vaccination, and more often had second and third seizures reported after subsequent vaccinations than other children.

Both short or prolonged, generalized or unilateral, and febrile or afebrile vaccination-related seizures occurred in children with *SCN1A*-related Dravet syndrome. Seizures occurred more often with a body temperature below 38.5°C, illustrating the high sensitivity^28^ also to minor temperature increase in children with *SCN1A*-related Dravet syndrome.

### Strengths and limitations of the study

This study is, as far as we could retrieve, the largest follow-up study on the prevalence of Dravet syndrome among children with seizures following vaccinations. The response rate in our study was high, especially among the parents of children diagnosed with epilepsy at stage 1. The long follow-up time enabled us to exclude Dravet syndrome with high certainty in the majority of cases. We have studied only the prevalence and clinical characteristics of children with clinical Dravet syndrome (including borderline SMEI) caused by *SCN1A*-mutations, and have not studied cases with Dravet syndrome caused by mutations in other genes, because these latter patients probably have different clinical characteristics, including the relation between seizures and vaccinations.

Cases with milder phenotypes (for example Genetic Epilepsy Febrile Seizures plus) related to *SCN1A*-mutations were not studied, because these phenotypes are variable and aspecific, the prognosis is more favorable and the chance of detecting a *SCN1A*-(missense) mutation in children with these phenotypes is low (10% in case of positive family history).[29]–[31]

At stage 1 of the study, the cohort was selected with data from a passive surveillance system with a stable rate of reported seizures over the years. With an active survey among ≈40,000 Dutch infants, the incidence rate was similar.[32] The rate was only slightly lower than reported in a RCT including DTwP.[32], [33] The report of the AEFI was delayed several years in some children with Dravet syndrome or other epilepsies, which suggests selective reporting of events that are followed by (severe) epilepsy, especially among delayed notifications. However, when we excluded AEFI that were reported more than two months after vaccination, the proportion of children with Dravet syndrome was even slightly higher.

At stage 1 of the study, we selected children who possibly had Dravet syndrome based on available data, out of the total cohort. Only of these children follow-up was obtained at stage 2. This selective follow-up, may have resulted in underdetection of cases with Dravet syndrome in the total cohort. However, the criteria for possible Dravet syndrome at stage 1 were broad, which makes inaccurate exclusion of children with Dravet syndrome unlikely.[28], [34]

Unfortunately, on few patients we were not fully informed or they were lost to follow-up. Therefore, the total proportion of *SCN1A*-related Dravet syndrome among children with seizures following vaccination, might be slightly higher. However, based on an estimated prevalence of Dravet syndrome of 1∶20,000 to 1∶40,000,[8]–[10] a proportion of vaccination-related seizures in children with Dravet syndrome of 27%,[17] and a proportion of detectable *SCN1A*-mutations of 70%, [12] the expected number of *SCN1A*-related Dravet syndrome among children with seizures following vaccination would be between 9 and 19 in this ten-year birth cohort of 1,97 million live births.[35] This is in agreement with our results.

In none of the children reported with seizures related to MMR-vaccinations, Dravet syndrome was diagnosed. There may be several explanations for this. Firstly, children with Dravet syndrome often have weekly seizures after one year of age, so the relationship with an MMR-vaccine administered a week before, will not always be recognized. In our cohort, at least two of the children with Dravet syndrome had seizures within the at risk time interval after MMR-vaccination but these were not reported. Secondly, children with Dravet syndrome were less often vaccinated with MMR-vaccine, only 35% (5 of 14) in this study (data not shown). Thirdly, the incidence of febrile seizures in the second year of life is higher, as is the incidence of fever after MMR-vaccinations (compared with the incidence after inactivated DTP-IPV-Hib vaccines), resulting in a higher frequency of (febrile) seizures after MMR-vaccinations in the general population (Figure 1).[3]

### Comparisons with previous studies and findings

No prospective studies on the prevalence of *SCN1A*-related Dravet syndrome among children with vaccination-related seizures have been published yet. A much smaller, retrospective study among children reported with suspected vaccination-related seizures under age six years, showed that 1.2% (4 out of 328) had *SCN1A*-related Dravet syndrome.[36] The overall results of that study are comparable with ours. However, the number of reported seizures in that study was 5-times lower when adjusted for population size, and the proportion of children diagnosed with epilepsy was higher. This suggests that in that study severe adverse events, or events which were followed by epilepsy, were more likely to be reported. Only in part of all children with clinical characteristics of Dravet syndrome, *SCN1A*-analysis was performed. Together, this resulted in a similar proportion of established *SCN1A*-related Dravet syndrome as in our study.

In the 12 children with Dravet syndrome with epilepsy onset after vaccination in our cohort, the mean age at first seizure was 4.1 months. These results are comparable with three previous studies showing a mean age of 4.0, 4.2 and 5.4 months, respectively, in Dravet syndrome patients with seizure onset after vaccination.[7], [10], [17] All of our Dravet syndrome patients had a seizure within 4 to 21 hours after vaccination with DTP-IPV(-)Hib vaccine, except for one in whom the seizure occurred 17 days later and was considered to be unrelated to the vaccination. In other studies, seizures occurred within 24 hours, or within 48 hours after administration of inactivated vaccines,[7], [37] but more exact time intervals were not reported. Also for the general pediatric population an increased seizure frequency has been reported only within the first 24 hours after administration of an inactivated vaccine.[3]

Although initial seizures in Dravet syndrome patients are often febrile, 57.9% of the seizures in our patients occurred with a temperature below 38.5°C. In the study of Berkovic et al, half of the seizures following vaccination were afebrile (defined as a body temperature below 38°C).[7] In another study one third of seizures following vaccination occurred without fever (definition of fever not reported).[17] These results emphasize that in patients with Dravet syndrome, even a mild increase in body temperature or an immune response without increased temperature, can be sufficient triggers for seizures. Moreover, this suggests that although in the general population there is no increased risk of afebrile seizures after inactivated vaccines,[3] this may be the case in subgroups of children with an increased seizure susceptibility.

### Unanswered questions and future research

The DTP-IPV vaccines that were administered during the ten-year cohort of our study consisted of a whole cell pertussis (wP) component in children vaccinated in the first eight years of the cohort, and an acellular pertussis (aP) component in the last two years.[38] In several countries wP-vaccines are still used, but the majority of developed countries adopted aP-vaccines.[39] The number of reported AEFI dropped after introduction of DTaP-IPV-Hib vaccine in 2005 in the Netherlands.[38] Interestingly, all detected cases of *SCN1A*-related Dravet syndrome were reported after vaccination with wP-component (Table 3). Therefore, our results may not be representative for aP-vaccines. However, two of our cases also had seizures following vaccinations not containing pertussis (cases 3 and 12), and previously, seizures after aP-vaccines in Dravet syndrome have been described.[16], [37] When longer term follow-up data of a more recent cohort will be available, the relation between the aP-vaccines and Dravet syndrome can be studied in more detail.

For children with Dravet syndrome further studies are warranted to evaluate if adapted vaccination schedules and preventive measures are needed to protect them optimally against infectious diseases while minimizing the risk of seizures. For this purpose, it is important that physicians who make a diagnosis of Dravet syndrome in children who have earlier been reported with seizures following vaccination, report this back to the surveillance system. For the majority of children diagnosed with Dravet syndrome in our study, this had not been done.

### Conclusions and implications

Our study shows that although *SCN1A*-related Dravet syndrome is a rare disorder, 2.5% of reported seizures following vaccinations in the first year of life occurred in children with this disorder. In children with Dravet syndrome, vaccinations might induce the first and following seizures. Physicians and co-workers of immunization safety surveillance programs should have knowledge of the prevalence of Dravet syndrome among children reported with seizures following vaccinations, and of the discriminating seizure characteristics in this subgroup. This will promote earlier diagnoses of Dravet syndrome which is important for appropriate treatment and genetic counseling. Moreover, an early diagnosis will prevent parents and professionals from assuming that vaccination is the cause of the epilepsy, and will thereby promote faith and participation in immunization programs.

## Supporting Information

Table S1 **Vaccination schedules from 1997–2006.**
(DOC)

Table S2 **Follow-up results of children according to classification of seizures at stage 1.**
(DOC)

Table S3 **Clinical features of children with Dravet syndrome.**
(DOCX)
