# Supplementary material for: Prevalence of SCN1A-Related Dravet Syndrome among Children Reported with Seizures following Vaccination: A Population-Based Ten-Year Cohort Study
Source: PLoS One. 2013 Jun 6;8(6):e65758. doi: 10.1371/journal.pone.0065758 (PMC3675088; doi:10.1371/journal.pone.0065758)
Supplement: Table S1 — Vaccination schedules from 1997–2006. (DOC) [file pone.0065758.s001.doc]

**Supplementary table 1: Vaccination schedules from 1997-2006**

Table 1A Vaccination schedules of the NIP from 1997-1999

| Age | Injection 1 | Injection 2 |
| --- | --- | --- |
| 3 months | DTwP-IPV | Hib |
| 4 months | DTwP-IPV | Hib |
| 5 months | DTwP-IPV | Hib |
| 11 months | DTwP-IPV | Hib |
| 14 months | MMR |  |
| 4 years | DT-IPV |  |
| 9 years | DT-IPV | MMR |

Table 2B Vaccination schedules of the NIP from 1999-2006

| Age | Injection 1 | Injection 2^f^ |
| --- | --- | --- |
| 2 months | DTP-IPV^a,b,c^ | Hib |
| 3 months | DTP-IPV^a,b,c^ | Hib |
| 4 months | DTP-IPV^a,b,c^ | Hib |
| 11 months | DTP-IPV^a,b,c^ | Hib |
| 14 months | MMR | Men C^d^ |
| 4 years | DT-IPV | aP^e^ |
| 9 years | DT-IPV | MMR |

^a^ = from 2003 onwards DTP-IPV was mixed with Hib.

^b^ = from 2003 onwards children of whom at least one parent was born in a country where hepatitis B is moderately or highly endemic and children of whom the mother tested positive for Hepatitis B surface Antigen (HBsAg) received DTP-IPV-Hib-HepB.

^c^ = from 2005 onwards DTwP-IPV-Hib was replaced by DTaP-IPV-Hib.

^d^ = from 2002 onwards

^e^ = since November 2001, from 2006 onwards mixed with DT-IPV.

^f^ = from April 2006 onwards infants received 7 valent conjugated pneumococcal vaccine simultaneously with DTaP-IPV-Hib(-HepB) at 2, 3, 4 and 11 months.
