# Supplementary material for: Prevalence of SCN1A-Related Dravet Syndrome among Children Reported with Seizures following Vaccination: A Population-Based Ten-Year Cohort Study
Source: PLoS One. 2013 Jun 6;8(6):e65758. doi: 10.1371/journal.pone.0065758 (PMC3675088; doi:10.1371/journal.pone.0065758)
Supplement: Table S2 — Follow-up results of children according to classification of seizures at stage 1. (DOC) [file pone.0065758.s002.doc]

**Supplementary table 2: Follow-up results of children according to classification of seizures at stage 1**

| Number of children according to classification of seizures in stage 1 | Single seizure | | Multiple seizures, classification | | | | Total |
| --- | --- | --- | --- | --- | --- | --- | --- |
|  | atypical seizure | seizure* | atypical seizures | febrile seizures | not further classified** | Epilepsy |  |
|  |  |  |  |  |  |  |  |
| Stage 1 |  |  |  |  |  |  |  |
| - Seizures reported after vaccinations | 505 | 375 | 108 | 163 | 21 | 97 | 1269 |
| **- *SCN1A*-related Dravet syndrome** | 0 | 0 | 0 | 0 | 0 | **2** | **2** |
| - Other etiological diagnosis | 3 | 4 | 1 | 3 | 1 | 13 | 25 |
| - No etiological diagnosis | 502 | 371 | 107 | 160 | 20 | 82 | 1242 |
| - Dravet syndrome possible | 63 (12.5%) | 84 (22.4%) | 4 (3.7%) | 77 (47.2%) | 8 (38.1%) | 43 (44.3%) | 279 (22.0%) |
|  |  |  |  |  |  |  |  |
| Stage 2 |  |  |  |  |  |  |  |
| - Follow-up data available | 49 (77.8%) | 70 (83.3%) | 3 (75.0%) | 67 (87.0%) | 5 (62.5%) | 40 (93.0%) | 234/279 (83.9%) |
| - Multiple seizures | 7 (14.3%) | 23 (32.9%) | - | - | - | - | 30/119 (25.2%) |
| - Diagnosed with epilepsy | 3 (6.1%) | 3 (4.3%) | 1 (33.3%) | 6 (9.0%) | 3 (60.0%) | - | 16/194 (8.2%) |
| **- *SCN1A*-related Dravet syndrome** | **1 (2.0%)** | **0** | **0** | **2 (3.0%)** | **2 (40.0%)** | **8 (20.0%)** | **13/234 (5.6%)** |
| - Other etiological diagnosis | 0 | 4 (5.7%) | 0 | 3 (4.5%) | 0 | 8 (20.0%) | 15/234 (6.4%) |
| - Inconclusive for Dravet syndrome | 1 (2.0%) | 0 | 0 | 0 | 0 | 1 (2.5%) | 2/234 (0.9%) |
|  |  |  |  |  |  |  |  |

* afebrile, simple febrile, and complex febrile seizures combined; ** combination of atypical, afebrile or febrile seizures.
