# Supplementary material for: Prevalence of SCN1A-Related Dravet Syndrome among Children Reported with Seizures following Vaccination: A Population-Based Ten-Year Cohort Study
Source: PLoS One. 2013 Jun 6;8(6):e65758. doi: 10.1371/journal.pone.0065758 (PMC3675088; doi:10.1371/journal.pone.0065758)
Supplement: Table S3 — Clinical features of children with Dravet syndrome. (DOCX) [file pone.0065758.s003.docx]

**Supplementary Table 3.** **Clinical features of children with Dravet syndrome.**

|  | M/F | Age at onset of seizures | Seizure types | Development delay | Neurological symptoms | Vaccination scheme | Age at DNA diagnosis | *SCN1A*-mutation | Ref |
| --- | --- | --- | --- | --- | --- | --- | --- | --- | --- |
|  |  |  |  |  |  |  |  |  |  |
| 1 | M | 5 mo | GTCS, H, SE, M, F, | ID | Autism, ataxia, hemiplegia | Incomplete: several vaccinations and without pertussis component | 8 years | p.Asp1239Tyr | [1] |
|  |  |  |  |  |  |  |  |  |  |
| 2 | M | 4 mo | GTCS, H, SE, A, M, | Mild ID  (IQ 71 at 7 y) |  | Stopped | 2 years | p.Leu1717Pro | - |
|  |  |  |  |  |  |  |  |  |  |
| 3 | F | 4 mo | GTCS, SP, SE, M, A, H, At | Moderate ID (TIQ 50 at 6 y) | Ataxia | Incomplete: without pertussis component | 8 years | p. Phe1535fs | - |
|  |  |  |  |  |  |  |  |  |  |
| 4 | F | 3.5 mo | GTCS, H, SP, CPS, A, M | Severe ID |  | Complete | 5 years | p.Gly1433Glu | [2] |
|  |  |  |  |  |  |  |  |  |  |
| 5 | M | 4 mo | GTCS, GT, SE, CPS | Delayed |  | Complete | 3 years | p.Arg946His | [3]** |
|  |  |  |  |  |  |  |  |  |  |
| 6 | M | 3 mo | GTCS, SE, M, A, CPS | 4 y DQ<50 | Autism, ataxia, hypotonia | Incomplete: one vaccination | 11 years* | p. Arg101Gln | [4]** |
|  |  |  |  |  |  |  |  |  |  |
| 7 | M | 4 mo | GTCS, M, Ast, A, SE | Mild |  | Incomplete: several vaccinations | 5 years* | duplication exon 17-20 | - |
|  |  |  |  |  |  |  |  |  |  |
| 8 | M | 5 mo | GTCS, H, GT, SE, M, CPS, A | Severe |  | Incomplete: several vaccinations | 3 years | c.4278_4282delCAAGT | - |
|  |  |  |  |  |  |  |  |  |  |
| 9 | F | 4.5 mo | GTCS, At, CPS, SE | Mild | Attention deficit | Incomplete: several vaccinations | 2 years | p.Val1390Met | [2]** |
|  |  |  |  |  |  |  |  |  |  |
| 10 | F | 3.5 mo | GTCS, SE, H, CPS | Moderate-severe |  | Incomplete: one vaccination | 2 years | c.3880-1G>A | [5] |
|  |  |  |  |  |  |  |  |  |  |
| 11 | M | 3 mo | GTCS, SE, H, A, SP | Delayed |  | Stopped | 1 years | p.Trp384Arg | - |
|  |  |  |  |  |  |  |  |  |  |
| 12 | M | 4.5 mo | GTCS, H, A, M | Severe ID |  | Incomplete: without pertussis component | 23 years | p.Ser1879fs | - |
|  |  |  |  |  |  |  |  |  |  |
| 13 | M | 6 mo | GTCS, H, GT | Moderate ID | Autism | Incomplete: without pertussis component | 13 years | p.Gly177Glu | [6]** |
|  |  |  |  |  |  |  |  |  |  |
| 14 | F | 4 mo | GTCS, CPS, A, M | Moderate-severe | Autism | Incomplete: without pertussis component | 11 years | p.Leu331fs | - |
|  |  |  |  |  |  |  |  |  |  |
| 15 | M | 5 mo | GTCS, H, SE, CPS, A, M | Severe | Autistic, ataxia | Stopped | 8 years | c.2176+2T>A | - |
|  |  |  |  |  |  |  |  |  |  |

A = (atypical) absences, At = atonic seizure, Ast = Astatic seizure, CPS = complex partial seizure, GTCS = Generalized tonic-clonic seizure, GT = Generalized tonic seizure, H = hemiconvulsions, M = myoclonus, SE = status epilepticus, SP = simple partial seizure.

Ref = references of previously described patients with identical mutations.

* Age at first referral for DNA diagnostics was younger, but only after re-analysis (case 6) or use of additional techniques (case 7) a SCN1A-mutation was detected.

** Mutation has been described in several other publications as well.

**References**

1. Depienne C, Trouillard O, Saint-Martin C, An I, Bouteiller D, et al. (2009) Spectrum of SCN1A gene mutations associated with dravet syndrome: Analysis of 333 patients. Journal of Medical Genetics 46: 183-191. 10.1136/jmg.2008.062323.

2. Sun H, Zhang Y, Liang J, Liu X, Ma X, et al. (2008) Seven novel SCN1A mutations in chinese patients with severe myoclonic epilepsy of infancy. Epilepsia 49: 1104-1107. 10.1111/j.1528-1167.2008.01549_2.x.

3. Berkovic SF, Harkin L, McMahon JM, Pelekanos JT, Zuberi SM, et al. (2006) De-novo mutations of the sodium channel gene SCN1A in alleged vaccine encephalopathy: A retrospective study. Lancet Neurology 5: 488-492. 10.1016/S1474-4422(06)70446-X.

4. Fukuma G, Oguni H, Shirasaka Y, Watanabe K, Miyajima T, et al. (2004) Mutations of neuronal voltage-gated na+ channel alpha 1 subunit gene SCN1A in core severe myoclonic epilepsy in infancy (SMEI) and in borderline SMEI (SMEB). Epilepsia 45: 140-148.

5. [Anonymous]. (2011) The SCN1A variant database. 04-01-2013.

6. Nabbout R, Gennaro E, Dalla Bernardina B, Dulac O, Madia F, et al. (2003) Spectrum of SCN1A mutations in severe myoclonic epilepsy of infancy. Neurology 60: 1961-1967.
